# A systematic review of the best-practice return to play programs in tennis players

**DOI:** 10.1371/journal.pone.0317877

**Published:** 2025-03-19

**Authors:** Adrián Martín-Castellanos, Manuel Barba-Ruiz, Iván Herrera-Peco, María Soledad Amor-Salamanca, Eva María Rodríguez-González, Francisco Hermosilla-Perona

**Affiliations:** 1 Department of Physical Activity and Sports Science, Alfonso X El Sabio University, Madrid, Spain; 2 Health Sciences Faculty, Alfonso X El Sabio University, Madrid, Spain; 3 Faculty of Health Sciences-HM Hospitals, University Camilo José Cela, Urb. Villafranca del Castillo, 49. Villanueva de la Cañada, Madrid, Spain; 4 Instituto de Investigación Sanitaria HM Hospitales, Madrid, Spain; 5 Facultad de Ciencias de la Vida y la Naturaleza, Universidad Nebrija, Madrid, Spain; Università degli Studi di Milano: Universita degli Studi di Milano, ITALY

## Abstract

Tennis is one of the most practiced sports in the world and with high injury rates across professional players and effective strategies to return to sport after an injury are necessary. Thus, this systematic review aims to assess and identify the most effective and evidence-based protocols developed during the return to play process in tennis players. The search was conducted using Web of Science, PubMed, and Scopus electronic databases. Studies that report a structure training program after suffering an injury in tennis players published before October 12, 2023, were identified. A total of 1164 studies were identified, after removal of duplicates and assess full text for eligibility 5 studies were included in the systematic review. RTP (return-to-play process) should be divided in three phases. Firstly, training should be focused on restoring ROM through mobility exercises, the inclusion of technical training are also available. Following, prioritize reaching pre-injury strength levels, integrating more technical training increasing hitting velocity. Finally, include serve technique while gradually increasing velocity. Trainers and coaching staff professionals’ needs consider that the duration and progression of RTP should be tailored to the individual characteristics of each player.

## Introduction

Tennis, recognized as one of the world’s most popular sports, owes its universal appeal with a mix of aerobic and anaerobic elements that cater to individuals of all ages and skill levels [1]. At the competitive level, tennis unveils a dynamic movements marked by the exchange of strokes and serves. Nevertheless, the sport’s demanding physical requirements could result in a risk of various musculoskeletal injuries for athletes [2]. While the specific occurrence of injuries varies based on factors like age, gender, and experience, studies encompassing the tennis community have shown that injury rates can range from 0.05 to 2.9 injuries per player annually [2]. Recent research on professional tennis competitions has revealed that injuries account for more than half of the withdrawals from both men’s and women’s events [3–5]. This prevalence of injuries has been analysed by numerous investigating the influence of tennis mechanics on the characteristics of different musculoskeletal injuries [6]. Descriptive epidemiological studies have indicated that injuries occur most frequently in the lower extremity, followed by the upper extremity, and lastly, the trunk [2,3,7]. However, lower extremity injuries in tennis are mostly acute and primarily result from trauma, while upper extremity injuries are predominantly chronic and stem from excessive and repetitive use [6].

To restore athletes to their preinjury performance levels coaches and physical trainers aid in attaining various physical function objectives. These include the restoration of complete range of motion, enhancement of strength, endurance, and power, as well as the reacquisition of dynamic stabilization, neuromuscular control, and sport-specific skills [2,8]. The selection of treatments and exercises is optional, with a focus on tailoring the rehabilitation plan to address an athlete’s specific needs, capabilities, and objectives play a pivotal role in the RTP (return-to-play process) [9].

The efficiency of the RTP often involves a combination of cardiovascular fitness, strength and flexibility exercises, as well as sport-specific drills to rebuild muscle coordination and enhance overall performance [10,11]. Efficient return to play training not only accelerates the recovery timeline but also reduces the risk of re-injury [12]. A well-structured program considers the individual player’s needs, injury history and the demands of the sport [10]. It is crucial to strike a balance between intensity and gradual progression to avoid overexertion and setbacks. A well-executed program not only facilitates a swift return to the court but also contributes to the long-term success and well-being of the athlete [13].

Nowadays, the scientific knowledge presents a substantial gap in our understanding of the dynamics and factors that influence an athlete’s safe and effective return to competitive tennis post-injury. The inadequacy of well-documented, evidence-based research restricts our ability to establish refined, effective, and tailored training programs for athletes and coaches, impede the refinement and standardization of RTP protocols and potentially prolonging recovery timelines and the susceptibility to reinjury. In this sense, a review of the training strategies developed to provide a safe recovery and effective return to competitive tennis post-injury has become an essential aspect that should be developed. Thus, the aim of this systematic review was to assess and identify the most effective and evidence-based protocols developed during the return to play process in tennis players.

## Methods

### Search strategy

The current systematic review was conducted following the guidelines provided in the Preferred Reporting Items for Systematic Reviews and Meta-Analyses (PRISMA) statement [14]. A comprehensive search of three online databases (PubMed, Scopus, Web of Science) was undertaken by two independent reviewers. The protocol was registered in the International Prospective Register of Systematic Reviews (PROSPERO) database.

Title, abstract and keyword fields were searched using the following search strategy: tennis AND injury OR injuries AND “return to play” AND “training program” OR “conditioning program”.

### Eligibility criteria

Studies were selected according to the inclusion and exclusion criteria established. Firstly, the inclusion criteria follow during the screening of the articles were: (i) articles published in English language, (ii) experimental research and case studies. Secondly, articles were retracted from the first search according to the following exclusions criteria: (i) publications that do not qualify as original articles, case studies, or reviews, (ii) articles focused on other sports, (iii) articles focused on other racquet sports, (iv) participants older than 45 years old and younger than 13 years old, (v) articles not focusing on structured training programs for return-to-play in injured tennis players.

### Study selection

Searches were limited to articles written in English language. Two researchers performed independently October 12, 2023, the identification, screening, eligibility, and inclusion of studies, with disagreement settled by a third researcher. Where abstracts suggested that papers were potentially suitable, the full-text versions were obtained and included in the review if they were found to fulfil the selection criteria. Reference lists of included papers and known published systematic reviews were hand searched to ensure the inclusion of all the available published evidence.

### Identification of studies

**Fig 1:**
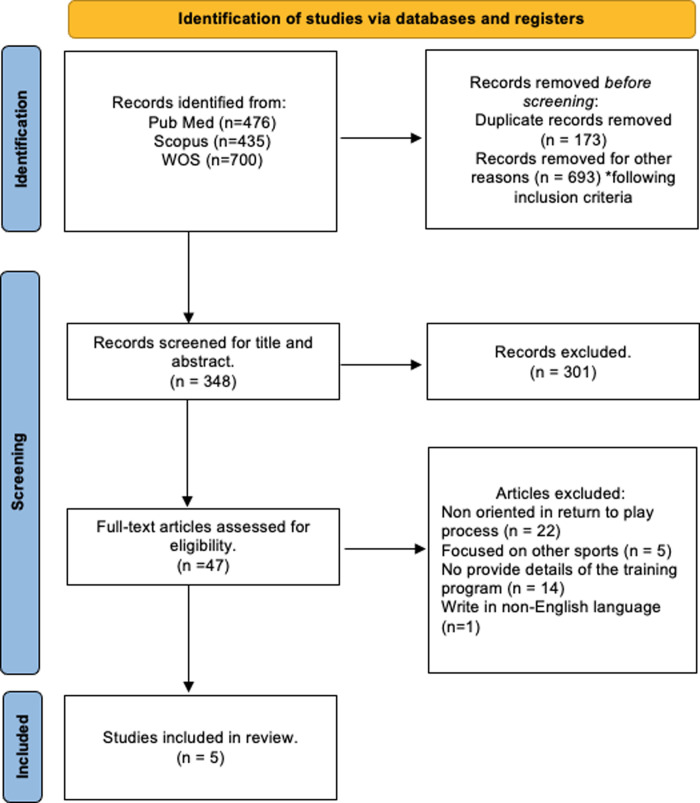
PRISMA flow diagram.

Our initial search identified a total of 1164 studies (see Fig 1 Flow diagram). The reference list of selected manuscripts was also examined for other potentially eligible manuscripts. After removal of duplicates and elimination of papers based on title and abstract screening, 348 manuscripts remained, 48 was assess full text for eligibility. Finally, 5 studies were included in the systematic review. The studies that did not match the eligibility criteria based on full-text screening were discarded for one or more of the following reasons: (i) articles not oriented in return to play process, (ii) articles focused on other sports, (iii) articles which do not provide details of the training program and (iv) articles written in non-English language.

### Data extraction

Two of the authors independently extracted characteristics of training protocols and results using a standardized form. Data extracted from all the eligible studies were injury type, main exercise classification, complementary exercise, recommended drills, exercise program. Based on the analysis of the contents of the reviewed publications and considering the RTP processes carried out in other sporting environments, the data were organised in 3 different phases: (i) Return to participation, (ii) Return to sport and (iii) Return to performance [15]. These phases were designed as a general guideline for applying rehabilitation and training across different sports. Typically, a continuum is followed that includes: (i) a gradual onset of rehabilitation and training, (ii) progression towards return to sport, and (iii) a goal of achieving performance levels comparable to pre-injury levels.

### Assessment of methodological quality and risk of bias

Two independent reviewers analysed the quality of included studies using the modified Newcastle-Ottawa Quality Assessment Scale (NOS) and Oxford Levels of Evidence (Table 1) [16]. The Newcastle Ottawa scale [17] for cohort and case–control studies have been adapted to case report studies by removing items that relate to comparability and adjustment (which are not relevant to non-comparative studies) and retained items that focused on selection, representativeness of cases and ascertainment of outcomes and exposure. This tool was applied in several published systematic reviews with good inter-rater agreement [18,19]. Newcastle Ottawa scale modifications converge into eight items that can be categorised into four domains: selection, ascertainment, causality and reporting [20]. Oxford Level of Evidence scores range from 1a to 5, with 1a a systematic review of high-quality randomized controlled trials, and 5 an expert opinion [16].

**Table 1:** Quality assessment according to modified Newcastle-Ottawa Quality Assessment Scale (NOS) and Oxford’s Evidence levels.

Modified Newcastle-Ottawa Quality Assessment Scale (NOS)										Oxford’s Evidence levels
	Selection	Ascertainment		Causality				Reporting	Total	
	1	2	3	4	5	6	7	8		
Bennet et al., 2017	*	*	*	–	–	*	*	*	6	4
Félix et al., 2021	–	*	*	–	–	–	*	*	4	4
Reinold et al., 2002	–	*	*	–	–	–	*	*	4	4
Amrani et al., 2019	*	*	–	–	–	*	*	*	5	4
Wang et al., 2023	*	*	*	–	–	*	*	*	6	4
Mean									5	4

⎯ =  no; *  =  yes; 1. Does the patient(s) represent(s) the whole experience of the investigator (centre)?, 2. Was the exposure adequately ascertained?, 3. Was the outcome adequately ascertained? 4. Were other alternative causes that may explain the observation ruled out?, 5. Was there a challenge/rechallenge phenomenon?, 6. Was there a dose–response effect?, 7. Was follow-up long enough for outcomes to occur? 8. Is the case(s) described with sufficient details to allow other investigators to replicate the research or to allow practitioners make inferences related to their own practice?

## Results

### Participants

The included studies presented athletes with a mean age of 19.66 years and a standard deviation of 4.19 years. Only the level of two subjects was reported, indicating that they competed at a semi-professional level. The studies primarily focused on return-to-play protocols for shoulder injuries, with interventions targeting rotator cuff, acromioclavicular, and glenohumeral joint issues emerging as the most frequent. The times from operation to RTP process were equally disparate, ranging from 10 weeks postoperatively to 6 months.

### Stages of intervention

#### Return to participation.

The athlete’s first approach during the first two weeks could be placed in this phase. Exercise recommendations were geared towards mobility and technical drills [21–25]. Mobility work generally involved the shoulder joint [21,24], and technical development focused on simple technical tasks throughout the week, mainly forehand and backhand groundstrokes [22,23,25]. The number of repetitions per set starts at around 10 [22,25], increasing throughout the week to as many as thirty for some strokes. Supplementary strength-based exercise guidelines are found,[21,24] although this is not the main objective, they are performed at low intensity (<6/10 RPE (Rate of perceived execution). Some considerations on hitting can be found depending on the location of the injury [21], as shown in the notes to Table 2.

**Table 2:** Description of the considerations for return to play recommended by the articles for return to participation stage.

Weeks	Authors	Injury type	Main exercise classification	Complementary exercise	Recommended drills	Notes	Exercise program
1	Bennet et al., (2017)	Subacromial impingement syndrome	Mobility	Strength	Shoulder internal and external rotation Shoulder abduction Pectoralis stretch	Shoulder at neutral Open-can position. Up to 90° of abduction Hold for 30 seconds	2 * 20 each drill 2 * 20 3 * 1
	Félix et al., (2021)	GR	Technical		Warm up (M) Forehand groundstrokes (FH/GS) (W) FH/GS and backhand/GS (F) FH/GS BH/GS	Weeks 1–3 avoid hitting with open stance for all GS for upper extremity injuries and 4–6 weeks for lower ones. Warm up 5-10 min: mini tennis. 5-10 min for rest between drills	(M) 5–10 FH/GS (W) 20–15 FH/GS BH/GS (F) 25–20 FH/GS BH/GS
	Reinold et al., (2002)	GR	Technical		(M) FH and BH (W) FH and BH (F) FH and BH	10 min rest between series	(M) 12 FH, 8 BH, (M) 13 FH, 7 BH (W) 15 FH, 8 BH, (W) 15 FH, 7 BH (F) 2 * 15 FH, 10 BH
	Amrani et al., (2019)	Rotator Cuff	Technical		(M) GS (W) GS (F) GS	5 min rest between series 6-10% MV	25 GS-Rest-25 GS 30 GS-Rest-30 GS 40 GS-Rest-40 GS
	Wang et al., (2023)	Rotator Cuff	Mobility	Strength	AAROM D1 Shoulder pendulum AROM Flexion Scapular squeeze AROM Scaption Isometric external and isotonic internal rotation Elbow extension Serratus punch	(<6/10 RPE) Isometric external rotation is performed with a towel roll; isotonic with a 2 kg band	3 * 15; Dowel 3 * 15; Table 3 * 15; N/A 3 * 15; N/A 3 * 15; N/A 3 * 15 * 5s 3 * 15; 2 kg band 3 * 15; N/A.
2	Bennet et al., (2017)	Subacromial impingement syndrome	Mobility	Strength	Shoulder internal and external rotation Shoulder abduction Pectoralis stretch	Shoulder at neutral Open-can position. Up to 90° of abduction Hold for 30 seconds	2 * 20 each drill 2 * 20 3 * 1
	Félix et al., (2021)	GR	Technical		(M) FH; BH (W) FH, BH (F) FH, BH	Weeks 1–3 avoid hitting with open stance for all GS for upper extremity injuries and 4–6 weeks for lower ones. 5-10 min for rest between drills	(M) 25–30 FH, 20–25 BH (W) 25–30 FH, 20–25 BH (F) 30–35 FH, 25–30 BH
	Reinold et al., (2002)	GR	Technical		(M) FH and BH (W) FH and BH (F) FH and BH	10 min rest between series	(M) 2 * 25 FH, 15 BH (W) 2 * 30 FH, 20 BH (F) 2 * 30 FH, 25 BH
	Amrani et al., (2019)	Rotator Cuff	Technical		(M) GS (W) GS (F) GS	5 min rest between series 10-16% MV	(M) 40 GS-Rest-40 GS (W) 55 GS-Rest-55 GS (F) 60 GS-Rest-40 GS Rest-30 GS
	Wang et al., (2023)	Rotator Cuff	Mobility	Strength	Flexion Resisted D1 Wall corner stretching Side-lying ER/IR Rhythmic stab Scaption Internal rotation Sleeper’s stretch Serratus punch	(<6/10 RPE)	3 * 15; 2 kg DB 3 * 15; 1 kg band 3 * 1 min; Wall 3 * 15; 2 kg DB Wall 3 * 15; 1 kg band 3 * 15; 3 kg band 3 * 1 min; N/A 3 * 15; 2 kg DB

GR =  Does not include type of injury, general recommendations; M =  Monday; W =  Wednesday; F =  Friday; S; Saturday; GS: Groundstroke FH =  forehand; BH =  backhand; FHV/BHV =  fore/back hand volley; SwV =  swinging volley; OH =  overhead; SR =  serve; MV = Match volume; AROM =  active range of motion; AAROM =  active assistive range of motion; DB =  dumbbell.

#### Return to sport.

In this phase, which covers the period from the 3rd week to the 6th week, the orientations towards exercise are eminently technical, supported by contents related to mobility and strength, being complementary aspects (Table 3).

**Table 3:** Description of the considerations for return to play recommended by the articles for return to sport stage.

Weeks	Authors	Injury type	Main exercise classification	Complementary exercise	Recommended drills	Notes	Exercise program
3	Bennet et al., (2017)	Subacromial impingement syndrome	Mobility	Strength	Shoulder internal and external rotation Shoulder abduction Pectoralis stretch Scapula push-ups Single arm row Lower trapezius activation	Shoulder at neutral Open-can position. Up to 90° of abduction Hold for 30 seconds. Initially with hands placed on a bench to ensure that the patient is not horizontal in order to reduce the difficulty. Shoulder at neutral Draw scapulae ‘down and back’. Hold for 5 seconds	2 * 20 each drill 2 * 20 3 * 1 3 * 10 3 * 10 2 * 10
	Félix et al., (2021)	GR	Technical		(M) FH; BH; shadow SR (W) FH; BH; shadow SR (F) SR Easy (<50% force), 10-min rest; FH; BH; FHV; BHV	Weeks 1–3 avoid hitting with open stance for all GS for upper extremity injuries and 4–6 weeks for lower ones. Volleys no fast velocity shots. Do not have your partner drill it at you	(M) 35–40 FH 30–35 BH 10–20 shadow SR; (W) 35–45 FH 35–45 BH 10–20 shadow SR; (F) SR Easy (<50% force), 10-min rest, 40–50 FH 40–50 BH 15–20 FHV 15–20 BHV
	Reinold et al., (2002)	GR	Technical		(M) FH, BH and SR (W) FH, BH and SR (F) FH, BH and SR	10 min rest between series	(M) 2 * 30 FH, 25 BH, 10 SR (W) 2 * 30 FH, 25 BH, 15 SR (F) 30 FH, 30 BH, 15 SR; 30 FH, 15 SR; 30 FH, 30 BH, 15 SR
	Amrani et al., (2019)	Rotator Cuff	Technical		(M) GS and SR (W) GS and SR (F) GS and SR	5 min rest between exercises 20-s rest between points 17-31% MV Serve speed 25% before injury	(M) 60 GS, 10 SR, Rest, 60 GS, 10 SR (W) 60 GS, 10 SR, Rest, 60 GS, 10 SR, Rest, 40 GS (F) 60 GS, 15 SR, Rest, Play 4 games, Rest, 40 GS, 15 SR
	Wang et al., (2023)	Rotator Cuff	Strength	Mobility	Flexion Resisted D1 Wall corner stretching Side-lying ER/IR Rhythmic stab Scaption Internal rotation Sleeper’s stretch Serratus punch	8–9/10 RPE	3 * 15; 2 kg DB 3 * 15; 1 kg band 3 * 1 min; Wall 3 * 15; 2 kg DB Wall 3 * 15; 1 kg band 3 * 15; 3 kg band 3 * 1 min; N/A 3 * 15; 2 kg DB
4	Bennet et al., (2017)	Subacromial impingement syndrome	Mobility	Strength	Shoulder internal and external rotation Shoulder abduction Pectoralis stretch Scapula push-ups Single arm row Lower trapezius activation Lower trapezius stability	Shoulder at neutral Open-can position. Up to 90° of abduction Hold for 30 seconds. Initially with hands placed on a bench to ensure that the patient is not horizontal in order to reduce the difficulty. Shoulder at neutral Draw scapulae ‘down and back’. Hold for 5 seconds Standing against a wall, abducting both arms to full 180° while keeping hands in contact to the wall and ensuring upper traps do not activate	2 * 20 each drill 2 * 20 3 * 1 3 * 10 3 * 10 2 * 10 3 * 10
	Félix et al., (2021)	GR	Technical		SR, ** Serves at 50% First Serve** FhV; BHV; SR; SwV; 10-min rest × 2	Continue increase in groundstrokes by 10–15 weekly Initiate advanced strokes, i.e., swinging volleys and slices if applicable to player’s skill level.	20–30 SR 15–20 FhV, 10–15 BHV, 30–35 SR, 20–25,10–15 SwV
	Reinold et al., (2002)	GR	Technical		(M) FH, BH and SR (W) FH, BH and SR (F) FH, BH and SR	A 10-minute break should be included between the first drills and subsequent games/ sets.	(M) 30 FH, 30 BH, 10 SR; 3 Games; 10 FH, 10 BH, 5 SR (W) 30 FH, 30 BH, 10 SR; 1 set; 10 FH, 10 BH, 5 SR (F) 30 FH, 30 BH, 10 SR; 1.5 set; 10 FH, 10 BH, 3 SR
	Amrani et al., (2019)	Rotator Cuff	Technical		(M) GS and SR (W) GS and SR (F) GS and SR	5 min rest between exercises 20-s rest between points 29-44% MV Serve speed 50% before injury	(M) 20 * 4-ball pts; Rest;15 SR; Rest; play 4 games; Rest; 15 SR (W) 20 * 4-ball pts; Rest;15 SR; Rest; play 6 games; Rest; 15 SR (F) 20 * 4-ball pts; Rest;15 SR; Rest; play 8 games; Rest; 15 SR
	Wang et al., (2023)	Rotator Cuff	Strength	Mobility	Cat-cow stretch Corner stretch Standing horizontal stretch Sitting IR Standing abduction Plank on elbows Knee push up No ball bat swing No ball serving	8–9/10 RPE	3 * 15; N/A 3 * 1 min; Wall 3 * 1 min; N/A 3 * 15; 2 kg band 3 * 15;2 kg DB 3 * 45 s; N/A 3 * 15; N/A 3 * 20; Tennis bat 3 * 20; Tennis bat
5	Bennet et al., (2017)	Subacromial impingement syndrome	Mobility	Strength	Shoulder internal and external rotation Shoulder abduction Pectoralis stretch Scapula push-ups Single arm row Lower trapezius stability	Shoulder at neutral Open-can position. Up to 90° of abduction Hold for 30 seconds. Starting with hands on a bench to reduce difficulty Shoulder at neutral Arms abduction to full 180°; keeping hands on the wall	2 * 20 each drill 2 * 20 3 * 1 3 * 10 3 * 10 3 * 10
	Félix et al., (2021)	GR	Technical		SR (1st serve 75%: 2nd serve 50%);10-min rest Initiate 5–10 OH (easy < 50% effort)	Continue increase in groundstrokes by 10–15 weekly Continue to increase volleys by 10–15 weekly	30–40 SR - 1st serve 75%: 10–20: 2nd serve 50% 5–10 OH
	Amrani et al., (2019)	Rotator Cuff	Technical		(M) GS and SR (W) GS and SR (F) GS and SR (S) GS and SR	5 min rest between exercises 20-s rest between points 49-64% MV Serve speed 75% before injury	(M) 20 * 4-ball pts; Rest; play 10 games; Rest; 10 SR (W) 20 * 4-ball pts; Rest; 15 * 6-ball pts; Rest; 15 * 6-ball pts; Rest; 20 * 4-ball pts; Rest; 10 SR (F) 20 * 4-ball pts; Rest; 15 * 6-ball pts; Rest; 15 * 6-ball pts; Rest; 20 * 4-ball pts (S) 20 * 4-ball pts; Rest; play 15 games; Rest; 10 SR
	Wang et al., (2023)	Rotator Cuff	Strength	Mobility	Cat-cow stretch Corner stretch Standing horizontal stretch Sitting IR Standing abduction Plank on elbows Knee push up No ball bat swing No ball serving		3 * 15; N/A 3 * 1 min; Wall 3 * 1 min; N/A 3 * 15; 2 kg band 3 * 15;2 kg DB 3 * 45 s; N/A 3 * 15; N/A 3 * 20; Tennis bat 3 * 20; Tennis bat
6	Bennet et al., (2017)	Subacromial impingement syndrome	Mobility	Strength	Shoulder internal and external rotation Shoulder abduction Pectoralis stretch Scapula push-ups Single arm row Lower trapezius stability	Shoulder at neutral Open-can position. Up to 90° of abduction Hold for 30 seconds. Starting with hands on a bench to reduce difficulty. Shoulder deadlift Arms abduction to full 180°; keeping hands on the wall.	2 * 20 each drill 2 * 20 3 * 1 3 * 10 3 * 10 3 * 10
	Félix et al., (2021)	GR			SR (1st 85%, 2^nd^ 50%) OH Practice all strokes (depends of skill level) without apprehension Play 4 games	Continue increase in groundstrokes by 10–15 weekly	40–45 (1st SR), 20–30: (2nd SR); 10–20 OH
	Amrani et al., (2019)	Rotator Cuff	Technical		(M) GS and SR (W) GS and SR (F) GS and SR (S) GS and SR	5 min rest between exercises 20-s rest between points 70-100% MV Serve speed 10% before injury	(M) 20 * 4-ball pts; Rest; play 16 games; Rest; 10 SR (W) 25 * 4-ball pts; Rest; 20 * 6-ball pts; Rest; 20 * 6-ball pts; Rest; 25 * 4-ball pts; Rest; 10 SR (F) 25 * 4-ball pts; Rest; 20 * 6-ball pts; Rest; 20 * 6-ball pts; Rest; 25 * 4-ball pts (S) 15 * 4-ball pts; Rest; play 24 games; Rest; 10 SR
	Wang et al., (2023)	Rotator Cuff	Strength	Mobility	Cat-cow stretch Corner stretch Standing horizontal stretch Sitting IR Standing abduction Plank on elbows Knee push up No ball bat swing No ball serving		3 * 15; N/A 3 * 1 min; Wall 3 * 1 min; N/A 3 * 15; 2 kg band 3 * 15;2 kg DB 3 * 45 s; N/A 3 * 15; N/A 3 * 20; Tennis bat 3 * 20; Tennis bat

GR =  Does not include type of injury, general recommendations; M =  Monday; W =  Wednesday; F =  Friday; S; Saturday; GS: Groundstroke FH =  forehand; BH =  backhand; FHV/BHV =  fore/back hand volley; SwV =  swinging volley; OH =  overhead; SR =  serve; MV = Match volume; AROM =  active range of motion; AAROM =  active assistive range of motion; DB =  dumbbell.

Regarding the recommended technical exercises, the number of repetitions per exercise should be increased in the previously included strokes (forehand and backhand) [22,23,25]. The serve also could be included in this stage [22,23,25], however, some indicators should be considered, as: at the beginning, serve must be executed with ranges of less than 50% of the athlete’s strength or at less than 25% of the athlete’s pre-injury speed, to increase the power week by week [22,23].

Similarly, from the third week onwards, volleys can be started [22], but at reduced speeds, increasing the pace progressively. Overhead strokes would be included during the fifth week, progressing in the sixth week. Some of the recommendations from the fifth week [22] onwards are based on hitting in a semi-closed and open stance, emphasising the continuation of strokes, and initiating high and low shots, high balls, all shots hitting crosscourt, and down centre line strokes. This phase would be orientated to the recovery of the athlete and the beginning of the search for their pre-injury levels.

Considering the mobility side, a higher number of drills are included, focussing on range of motion and stability work for the shoulder and upper extremity [21,24]. The proposals should follow a progression, facilitating the exercise at the beginning (positions from the wall or facilitating the demands of the position of the athlete who performs it) to progress according to his evolution [21].

Strength exercises aimed primarily at improving the upper body include an increasing perceived exertion rate (8-10 RPE) [24], although that in the early stages adaptations are recommended, for example, scapula push-ups offer the consideration of performing the exercise on a bench to ease the difficulty [21]. These recommendations are repeated throughout this phase, although some authors include core work and discourage service strokes. However, this may depend on the degree and type of injury.

Thus, in this phase, the aim is to progressively increase the load with aspects related to the athlete’s performance context; therefore, the volume of training sessions or series/repetitions increases in this section [23]. The inclusion of modified games is also considered, controlling the number of games and breaks. This progressive adaptation is proposed by some authors from week 4 onwards [22,23].

#### Return to performance.

**Fig 2:**
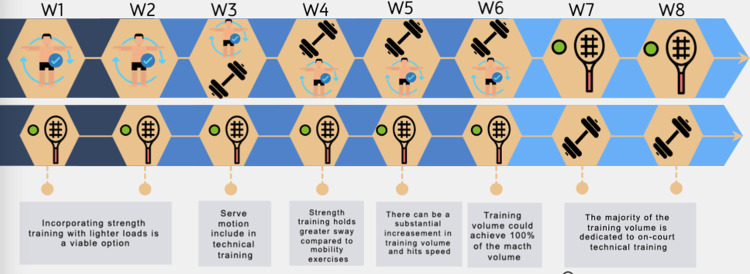
Primary training features based on each week. Note: [

In this last section, the athlete is approached towards his or her performance in the field. Factors related to strength and technical training come into play. The athlete performs serves at intensities close to 100%, which will increase until the eighth week, including the practice of conditioned simulated matches with breaks for rest (Table 4). These recommendations could also be modified according to the level of competition of the trained athletes, with more complex strokes not recommended for amateur athletes [22]. All these phases are resumed on the Fig 2.

**Table 4:** Description of the considerations for return to play recommended by the articles included in the research.

Weeks	Authors	Injury type	Main exercise classification	Complementary exercise	Recommended drills	Notes	Exercise program
7	Félix et al., 2021	GR	Technical		SR (1st 90%: 2nd 75%) OH Practice applicable strokes to skill level without apprehension Play simulated 1 set; taking rest breaks with every 3rd game	• Return to match play if no pain experienced in prior weeks (1st serve 90–95%, 2nd serve 75–80%) • Slowly work up towards 100% in each serve. Continue to practice all strokes (applicable to skill level)	40–45 (1st SR) 20 (2nd SR); 25–30 OH
	Wang et al., (2023)	Rotator Cuff	Strength	Technical	Corner stretch Standing horizontal stretch 50% intensity serving practice 75% intensity serving practice 50% ball contact stroke		3 * 1 min; Wall 3 * 1 min; N/A <80 × total for the ﬁrst 3 days <80 × total for the rest of the 1st week <180 × total for the week
8	Félix et al., 2021	GR	Technical		SR (1st and 2nd serve 100%) (M/W) Play simulated 1 set match following rules taking rest breaks with every 3rd game (F) Play simulated 2 set match following rules taking rest breaks with every 3rd game.	Modifications for Interval Tennis Program (ITP): Non-competitive players/amateurs (plays < 10 year) advance strokes not applicable, ITP completed in 4–5 weeks Competitive recreational players (USTA level > 3.0/juniors/collegiate and professional proceed with full ITP	

GR =  Does not include type of injury, general recommendations; M =  Monday; W =  Wednesday; F =  Friday; S; Saturday; GS: Groundstroke FH =  forehand; BH =  backhand; FHV/BHV =  fore/back hand volley; SwV =  swinging volley; OH =  overhead; SR =  serve; MV = Match volume; AROM =  active range of motion; AAROM =  active assistive range of motion; DB =  dumbbell.

**Figure:**
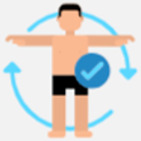

]:Mobility exercises. [

**Figure:**
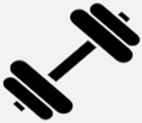

]:Strength training; [

**Figure:**
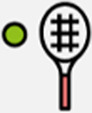

]: On court technical training.

The first line represents the main training, while the second line denotes the supplementary training. W represents each week.

## Discussion

The main objective of this research article was to examine the return to play training programs carried out by tennis players after suffering a musculoskeletal injury. However, one of the principal limitations to develop this research has been the limited number of articles in the field. Only five studies had developed different training programs focused and organised over the return to play in tennis players [21–25]. Despite this limitation, the research included in this review offers valuable insights for structuring training loads and exercises following a musculoskeletal injury. The research recommendations and findings presented here concentrate on post-shoulder injury rehabilitation, as the articles included are exclusively focused on this topic.

RTP is multifactorial and is dependent of several intrinsic and extrinsic factors, there are so many aspects that could be consider during the RTP as reestablishment of muscle strength and joint ROM (range of motion), regaining of proper motor control and proprioception, psychological readiness and recovery of functional ability [10]. Previous studies have analysed the return to play process in other sports as baseball [26,27]. Rebelo-Marques, Andrade (10) established different phases (return to participation, return to sport and return to performance) according to the training characteristics which can be applied also to tennis.

Firstly, the recommendations based on the studies analysed suggest that during the first two weeks after the rehabilitation process, the main exercises are developed with the aim of recovering full ROM with mobility exercises and initiating specific technical training. The focus should be on mobility as the main component of practice [21,22], and gradually include technical training, performing some specific drills such as forehand and backhand strokes [23–25], In this sense, traditional non-operative and post-operative rehabilitation programs for these athletes involve a gradual restoration of range of motion (ROM), strength, muscular endurance, dynamic stabilization and neuromuscular control [8,28].

Similarly, it has been observed that the intensity recommendations in the different studies should not be above 6 points over 10 in the Rating of Perceived Exertion (RPE) [24]. Besides, Amrani, Gallucci (23) indicate that the maximum volume during this two weeks should not be over 15% of the match volume. During the technical training Félix, Dines (22) noted that it could be include some technical movements (forehand and backhand), However, the overall recommendation from this study advises tennis players to avoid the hit with open stance for all groundstrokes for upper extremity during the first three weeks.

Upon successful completion of the early phases of the rehabilitation program, a gradual and controlled return to sport activities has been advocated by several authors [28–31]. In our results, this phase was developed during the following weeks until the 6 weeks. This phase in the research included in this review are characterised by the inclusion of strength training (core training can also be included) and technical training about the serve motion and volleys could be part of the training exercises [22,23]. The intensity raises until 8-10 RPE [24] and the training volume can achieve values around 70% of the match volume [23]. Strength training should achieve a balance between anterior and posterior shoulder musculature, special emphasis should be given to the posterior rotator cuff and scapular musculature for any strengthening program [8,28].

Beginning with the sixth week the players do the 100% of the volume training performed before the injury. Thus, it can be considered as the starting point of the return to performance phase that would finish when the athletes recover the preinjury specific performance. This phase could be characterised with the facts that executes serves with intensities nearing 100% could take part of the daily training and carrying out conditioned simulated match practices with intervals for rest [22,24].

Finally, it is crucial to underscore that the training program ought to be tailored to each individual’s specific needs and circumstances. The recommendations of the research articles include in this review needs to be adapted to each specific case, especially the temporal margins. Although models or decision-making guidelines are generated for the RTP process, this personalised approach ensures that the rehabilitation process effectively addresses the player’s strengths, weaknesses and recovery timeline, maximising their chances of a successful return to competition and reducing the re-injury probability [9,32].

## Conclusion

The tennis player’s return-to-play process should adhere to three stages tailored to their training needs. Initially, the focus is on restoring full range of motion (ROM) through mobility exercises, potentially incorporating technical training. Subsequently, the emphasis shifts to reaching pre-injury levels of strength, incorporating more technical training to enhance hitting velocity. Lastly, serve technique is addressed, with a gradual increase in serve velocity. However, each of these aspects should be personalized to suit the individual player’s characteristics.

## Supporting information

S1 File Prisma checklist. (DOCX)

S2 File Search database. (XLSX)
